# Cell and Molecular Mechanics in Health and Disease

**DOI:** 10.1155/2017/2860241

**Published:** 2017-02-13

**Authors:** Keiko Kawauchi, Hideaki Fujita, Daisuke Miyoshi, Evelyn K. F. Yim, Hiroaki Hirata

**Affiliations:** ^1^Frontiers of Innovative Research in Science and Technology, Konan University, 7-1-20 Minatojima-Minamimachi, Chuo-ku, Kobe, Hyogo 650-0047, Japan; ^2^Laboratory for Comprehensive Bioimaging, Riken Qbic, 6-2-3 Furuedai, Suita, Osaka 565-0874, Japan; ^3^Department of Chemical Engineering, University of Waterloo, 200 University Avenue West, Waterloo, ON, Canada N2L 3G1; ^4^Nagoya University Graduate School of Medicine, 65 Tsurumai, Showa-ku, Nagoya, Aichi 466-8550, Japan

A growing body of research has revealed that not only chemical stimuli, but also the mechanical stimuli in the microenvironment surrounding cells have a significant impact on cell behaviors. Mechanical stimuli from the environment are detected by various mechanosensing biomolecules including mechanosensitive ion channels and nonion channel type mechanosensors, leading to modulation of intracellular signaling. Notably, while subcellular structures—including cytoskeletons, membranous organelles, and the nucleus—influence and determine mechanical properties of cells, the mechanical properties of both cells and their microenvironment are altered in cancer or other diseases such as infections, chronic inflammation, and degenerative diseases. The abnormal expression and activation of mechanosensing biomolecules and/or components of the subcellular structures, which are sometimes accompanied by gene mutations, have been observed in many disease states. Such alterations potentially tune the chemical and physical interactions between cells and their environments and thereby contribute to disease progression. Therefore, the mechanical properties of individual subcellular structures (e.g., the actin cytoskeleton, cell adhesion complexes, and chromosomes) and the signaling molecules regulating these structures are emerging as targets in the research seeking mechanistic understanding of disease development or potential therapies for the diseases.

The original and review papers in this special issue provide novel insights into the mechanical cue-dependent regulation of cellular functions in various types of cells including bacterial cells, erythrocytes, fibroblasts, endothelial cells, and cancer cells. The topics range from the mechanosensing element at the plasma membrane to mechanical-induced intracellular signaling and structural remodeling and the mechanical modulation of the cancer therapy.

T. Nomura et al. addressed an inactivation mechanism of the mechanosensing biomolecule, MscS ion channel and revealed the contributions of positively charged residues in the channel molecule to the membrane potential-dependent inactivation of MscS.

Using numerical simulations, T. Wang et al. systematically examined how malaria infection-induced changes in mechanical properties of the erythrocyte membrane affect the blood flow in microvessels with stenosis. They showed that both deformability and adhesiveness of the erythrocyte membrane are critical factors characterizing the blood flow, which may provide the mechanical basis for microcirculatory obstruction in sever malaria.

D. Nobezawa et al. showed that, in contrast to the classical view, actin polymerization, rather than myosin II activity, plays a dominant role in driving the retrograde movement of the actin cytoskeleton at the protruding edge of a migrating cell.

Using detailed live-cell imaging, M. Sugawara et al. examined the mechanism that regulates the directional change in cell migration. They showed that, during the directional change, a migrating cell exhibits spatial coordination of three distinct dynamics of focal adhesion and the actin cytoskeleton, which potentially contributes to the change of cell polarity.

H. Takada et al. reported that while mechanical stretch of wounded skin accelerates the wound repair, this effect of mechanical stretch is enhanced by treating the skin with hyperforin, a major component of a traditional herbal medicine.

Several other outstanding papers showed and discussed that the physical properties of mechanical environments or mechanical stimuli alter the cell characters via intracellular signaling pathways including NF-*κ*B and p53 signaling pathways.

K. Suzuki and D. Yoshino reported that the treatment of endothelial cells with micropower plasma at gas-liquid interface promotes their proliferation. Furthermore, the plasma-induced NF-*κ*B activation is shown to be involved as underlying intracellular signaling.

The group of K. Kawauchi, who is one of the Editorial team members, showed that stiffness of the extracellular matrix (ECM) modulates the effect of chemotherapy on cancer cell growth. Soft ECM attenuates chemotherapeutic agent-induced activation of the tumor suppressor p53 in breast cancer cells, leading to cell growth resistance against chemotherapy.

In addition to original research papers, four interesting review papers discuss the relationship of mechanosensing and various diseases. K. Kawauchi's group reviewed the roles of the tumor suppressors p53 and retinoblastoma protein (pRb), whose signaling pathways are disrupted in many types of cancers, in the regulation of mechanosensing biomolecules and the actin cytoskeleton.

C. Uchida focused on the role of pRb in the regulation of chromatin structures. The paper reviewed how physical interactions of pRb with histone modifiers and chromatin factors control nucleosome/chromatin structures and discussed a potent strategy of cancer therapies that target these protein factors.

Y. Nakahata and Y. Bessho described an overview of the role of NAD+ metabolism in circadian changes of mechanical properties of chromosome/chromatin. Furthermore, they discussed the possibility that aging-associated diseases may be treated by interfering with NAD+ metabolism.

Y. Abe and N. Tanaka reviewed how the abnormal activation of the hedgehog signaling pathway, which plays a critical role in development and tissue homeostasis, leads to progression of lung cancer. In particular, they pointed out that physical interaction of non-small cell lung cancer (NSCLC) cells with cancer-associated fibroblasts enhances metastatic potential of NSCLC cells by activating the hedgehog signaling pathway.

The papers in this special issue illustrate that modulations of mechanosignaling pathways may provide novel approaches for development of new therapeutic methods which are distinctive from the conventional chemical therapies. The new discoveries will potentially lead to further in-depth studies on mechanochemical transduction mechanisms at molecular and supramolecular levels in the future.

*Keiko Kawauchi* *Hideaki Fujita* *Daisuke Miyoshi* *Evelyn K. F. Yim* *Hiroaki Hirata*

